# Association of the co-expression of SOX2 and Podoplanin in the progression of oral squamous cell carcinomas - an immunohistochemical study

**DOI:** 10.1590/1678-7757-2018-0348

**Published:** 2019-08-30

**Authors:** Sonali Pradhan, Vasudeva Guddattu, Monica Charlotte Solomon

**Affiliations:** 1 Manipal Academy of Higher Education Manipal College of Dental Science Department of Oral Pathology and Microbiology Manipal India Manipal Academy of Higher Education, Manipal College of Dental Science, Department of Oral Pathology and Microbiology, Manipal, India.; 2 Manipal Academy of Higher Education School of Public Health Department of Statistics Manipal India Manipal Academy of Higher Education, School of Public Health, Department of Statistics, Prasanna, Manipal, India.

**Keywords:** Sex determining region Y-box 2, Stem cell, Podoplanin, Invasion and metastasis, Oral squamous cell carcinoma

## Abstract

SOX2 is a transcription factor related to the maintenance of stem cells in a pluripotent state. Podoplanin is a type of transmembrane sialoglycoprotein, which plays an important role in tumor progression and metastasis. This study aims to determine association of SOX2 and podoplanin expression in the progression of oral squamous cell carcinomas and to elucidate the association between two proteins.

**Methodology:**

The immunohistochemical expression of SOX2 and podoplanin were evaluated in 60 cases of primary oral squamous cell carcinomas. The correlation between the SOX2 and podoplanin expression and the clinicopathological features of the tumors and the patient outcomes were assessed.

**Results:**

The expression of SOX2 was seen in 38/60 (63%) of the cases and the expression for podoplanin was seen in 45/60 (75%) cases. There was a significant inverse correlation between the expression of SOX2 and podoplanin with the tumor grade (p=0.002 and p=0.017, respectively). There was a high expression of SOX2 in 9/13 cases that presented with disease free survival. Survival analysis showed that a high expression of SOX2 correlated positively (p=0.043) with the disease-free survival. There was a significant positive association between the pattern of SOX2 and podoplanin expression (p=0.002).

**Conclusion:**

A high expression of SOX2 was associated with better disease-free survival. The expression of podoplanin was associated with the degree of differentiation of the tumors. Analysis of these biomarkers can aid in the prognosis and treatment of oral squamous cell carcinomas.

## Introduction

Oral squamous cell carcinoma is the sixth most common cancer reported globally. The annual incidence of oral squamous cell carcinomas is over 300,000 cases, of which 62% arise in developing countries.^1^ Though diagnostic techniques have improved, the survival rates are poor and have remained more or less the same for years, with a 5-year survival rate of approximately 50% cases from the time of diagnosis.^2^ Thus, there is a need to understand the proliferative activity, degree of differentiation, and the invasion and metastatic potential of the tumor.^3^

Sex determining region Y–related Homo box gene 2 (SOX2) is a transcription factor that is involved in maintaining stem cells in a pluripotent state.^4^ Increased nuclear reactivity is suggestive of embryonic dedifferentiation and the acquisition of stem cell properties of tumor cells.^5^ The role of SOX2 in oral squamous cell carcinoma is not widely explored and is still not precisely understood. A study evaluating the expression of SOX2 in oral squamous cell carcinomas has shown that a tumor with a high nuclear expression of SOX2 had longer disease-free survival period following postoperative radiotherapy.^5^ On the other hand, it has been shown that SOX2 was able to protect malignant cells from apoptosis throughout the journey of carcinogenesis.^6^

Podoplanin (PDPN) is a 162 amino acid, mucin-type of transmembrane sialoglycoprotein. It is named after its expression in renal podocytes of rats.^7^ Expression of podoplanin is rarely seen in normal oral mucosa, but is frequently seen in oral cancers. Podoplanin expression is evident in the early phases of oral squamous cell carcinoma transformation and it has been able to distinguish dysplastic lesion that transformed to malignancy from those that did not progress.^8^ It has been associated emphatically with tumor invasion and metastasis.^9^ Interestingly, Atsumi, et al.^10^ (2008) have shown that podoplanin-positive cancer cells exhibited stem cell-like properties, as they had the ability to undergo repopulation and give rise to heterogenous cancer cell population.^10^

Few studies have analyzed the expression of SOX2 and podoplanin in the progression of oral squamous cell carcinomas. These studies evaluated the role of the biomarkers separately.^11^^–^^15^ This study aimed to correlate the combined expression of SOX2 and podoplanin with the clinicopathological features of oral squamous cell carcinoma, and thereby to determine their influence on the progression and clinical outcome of oral squamous cell carcinomas.

## Methodology

The study was approved by the institutional ethics committee (IEC 656/2015). The material for the study comprised of archival formalin fixed paraffin embedded tumor blocks (FFPE) of primary oral squamous cell carcinoma cases (n=60). Twenty cases each of well differentiated, moderately differentiated and poorly differentiated tumors were retrieved from the departmental archives. Lymph nodes tissues sections and glioma tissue sections were positive controls for SOX2 and podoplanin and respectively.

### Patient selection

#### Inclusion criteria

Primary cases of oral squamous cell carcinoma in which treatment (chemotherapy or radiotherapy) had not begun

#### Exclusion criteria

Tumors that were not oral squamous cell carcinomas

Patients with systemic diseases

Immune-compromised patients

The clinical and follow up details of all the patients were obtained from their medical records. All the patients were followed up for a period of 6 months to 24 months. All biopsies specimens that were obtained prior to treatment were employed for this study. The follow-up data of the patients was available for only 30 patients, of which 13 were surviving free of the disease, 14 patients presented recurrence, and 3 patients had died of the disease.

### Immunohistochemistry

Sections of 4 µm thick obtained from the FFPE tissue blocks were taken onto slides coated with 3-aminopropyl triethoxysilane (APES, Sigma – Aldrich Co. St. Louis, USA). Sections were then deparaffinised. Antigen retrieval was performed using tris EDTA buffer at pH 9.0, Endogenous peroxide was neutralized by treating the sections with pre-diluted 3% hydrogen peroxide. Following, these sections were incubated with pre diluted primary antibody-SOX2 rabbit monoclonal antibody (SOX2-EP103, Pathnsitu, California, USA) and for podoplanin pre diluted primary antibody-Podoplanin rabbit monoclonal antibody (Podoplanin D2-40 Pathnsitu, California, USA) for 1 hour at room temperature. Then the sections were incubated with pre-diluted primary target binder (PolyExcel Target Binder, California PathnSitu, California, USA) at room temperature for 10 minutes. Slides were then incubated with secondary antibody (pre diluted PolyExcel Poly HRP, PathnSitu, California, USA), at room temperature for 10 minutes. The peroxidase activity was developed with diaminobenzidine tetrahydrochloride (DAB PathnSitu, California, USA,). Finally, the sections were counter stained with Mayer's haematoxylin, dehydrated, cleared and mounted with DPX (dibutyl phthalate xylene).

Lymph nodes and glioma tissue sections were positive controls for SOX2 and podoplanin, respectively. The negative control for the staining procedure was a tissue section of normal buccal mucosa for which the entire immunohistochemical procedure was carried out, except that the primary antibody was not used.

### Evaluation of SOX2 and podoplanin expression

The sections were observed under a light microscope (Olympus-BX21). A dark brown staining in the nucleus of epithelial cells was considered positive for the expression of SOX2, and in the cytoplasmic and/or membrane of epithelial cells was considered positive for podoplanin. The expression of the biomarkers was analyzed in a semi-quantitatively by two observers.

### Scoring system for SOX2

The immunohistochemical expression pattern of SOX2 was assessed according to the criteria given by Ge, et al.^16^ (2010), wherein the percentage of positive tumor cells was assessed. Five areas in the whole tumor sections were selected for each case. The percentages of positive tumor cells in all 5 fields were summed up and the mean of the percentages was recorded. Score 0=Negative Expression, Score 1 (Weak expression)=25% cells were positive, Score 2 (Moderate Expression)=26–50% cells were positive and Score 3 (Strong expression)=50% of cells were positive.

### Scoring system for podoplanin

The expression of podoplanin was evaluated by modifying the scoring system given by Yuan, et al.^15^ (2006). Five representative areas in the whole tumor sections were selected. The percentages of tumor cells with positive cytoplasmic/ membranous stain in the 5 fields were summed up and the mean of the percentages was recorded. Score 0=Negative Expression, Score 1 (Weak Expression)=1–10% positive cells, Score 2 (Moderate Expression)=11–50% positive cells, Score 3 (Strong expression)=51–100% positive cells.

**Figure 1:**
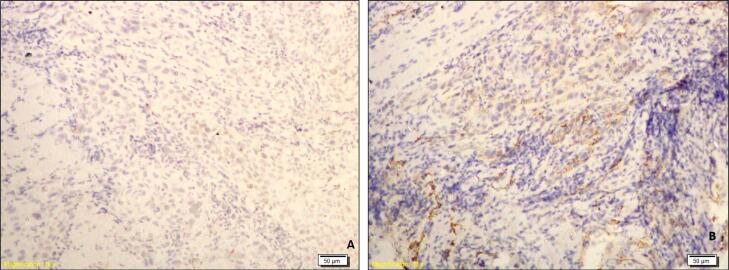
Photomicrograph showing a case of OSCC of Group 1 with low expression of SOX2 (A) and Low expression of podoplanin (B)

**Figure 2:**
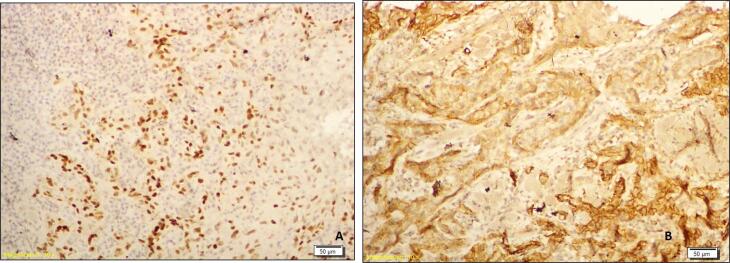
Photomicrograph showing a case of OSCC of Group 2 with a high expression of SOX2 (A) and a high expression of podoplanin (B)

**Figure 3:**
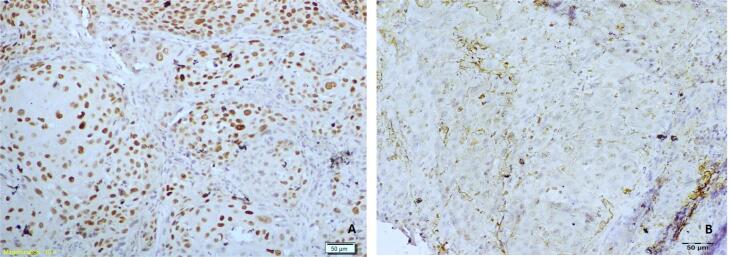
Photomicrograph showing a case of OSCC of Group 3 with high expression of SOX2 (A) and low expression of podoplanin (B)

**Figure 4:**
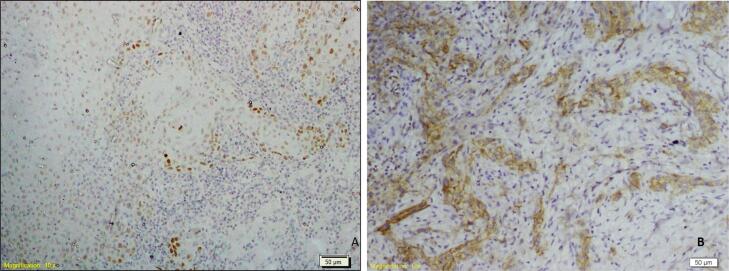
Photomicrograph and a case of OSCC of Group 4 with a low expression of SOX2 (A) and a high expression of podoplanin (B)

The cases were further categorized as those with a low expression (score 0 and score 1) and those with a high expression (score 2 and score 3) of SOX-2 and podoplanin. Further, the cases were categorized in 4 groups: Group 1 (There was a low expression of both SOX2 and podoplanin by the tumor cells – Figure 1A and Figure 1B), Group 2 (There was a high expression of both SOX2 and podoplanin by the tumor cells – Figure 2A and 2B), Group 3 (There was a high expression of SOX2 and low expression of podoplanin by the tumor cells – Figure 3A and Figure 3B) and Group 4 (There was a low expression of SOX2 and high expression of podoplanin by the tumor cells – Figure 4A and Figure 4B).

### Statistical analysis

All statistical analyses were performed using the Statistical Package for Social Sciences (SPSS) version 16.0. Descriptive analysis was carried out for patient characteristics. Inter-observer variability was assessed using Cohen's kappa coefficient and it was found to have good reproducibility (0.951). The association between clinicopathological parameters and the expression of SOX2 and podoplanin was assessed using the Chi-Square test. Survival analysis was carried out using Kaplan-Meier analysis and the Log-rank test. A p value of <0.05 was considered to be statistically significant.

## Results

The study group involved patients between the age group of 28 to 79 years old, with a mean age of 52.7±13.130 years. Among the 60 cases, 47/60 (78.3%) were males and 13/60(21.6%) were females. The most predominant site of the lesions was the tongue, wherein 22/60 (37%) of the tumors occurred.

In the study group, positive SOX2 expression in the nucleus of the tumor cells was observed in 38/60 (63.3%) cases. Among the 60 cases, 6/60 (10%) cases showed a weak expression (score 1) of SOX2, 7/60 (11.6%) cases showed moderate SOX2 expression (score 2) 25/60 (41.6%) cases showed strong SOX2 expression (score 3).

In the study group, positive podoplanin expression in the cell membrane/cytoplasm of the tumor cells was observed in 45/60 (75%) cases. Among the 60 cases, 16/60 (26.7%) showed a low expression (Score 1) of podoplanin, 8/60 (13.3%) cases showed moderate expression (Score 2), and 21/60 (35%) cases showed strong podoplanin expression (Score 3).

The association of the SOX2 and podoplanin expression with clinicopathological features when assessed as individual markers is given in Table 1. The expression of SOX2 correlated positively with the stage of the tumor (p=0.029) and inversely with the grade of the tumor (p=0.002). The expression of podoplanin correlated inversely (p=0.017) with the degree of differentiation of the tumor.

**Table 1:** Association of the expression of SOX2 and podoplanin with clinicopathological features and patient outcomes

		SOX-2 Expression			Expression of Podoplanin		
Clinico-pathological parameters	No of cases	Low (n=29)	High (n=31)	p Value	Low (n=31)	High (n=29)	p value
**Age**							
<40 years	11	5	6	0.549	4	7	0.215
>40 years	49	24	25		27	22	
**Gender**							
Male	47	21	26	0.223	22	25	0.132
Female	13	8	5		9	4	
**Habits**							
Tobacco habits	45	21	24	0.491	21	24	0.157
No habits	14	8	6		10	4	
Unknown	1	0	1		0	1	
**Site**							
Buccal mucosa	20	11	9	0.65	9	11	0.599
Tongue	22	9	13		11	11	
Gingiva/alveolus	18	9	9		11	7	
**Stage**							
Early stage	8	1	7	0.029*	3	5	0.296
Advanced stage	51	28	23		28	23	
Unknown	1						
**Grade**							
Well	20	8	12	0.002*	6	14	.017**
Moderately	20	5	15		10	10	
Poorly	20	16	4		15	5	
**Patient outcome**							
Disease free survival	13	4	9	0.093	4	9	0.183
Recurrence	14	6	8		9	5	
Death	3	3	0		2	1	

Follow-up details were not available for 30 cases, habit history and the stage of the tumor was not available for 1 case

Association of the combined expression of SOX2 and podoplanin with clinicopathological features and patient outcome is given in Table 2. The combined expression of both biomarkers correlated with the differentiation degree of the tumors (p=0.005). There was a significant association between the pattern of expression of the SOX2 and podoplanin (p=0.002) (Table 3).

**Table 2:** Association of the combined expressions of SOX2 and podoplanin with clinicopathological features and patient outcomes

Parameters	Number of cases	Low expression of SOX-2 and Low expression of podoplanin (Group 1) (n=20)	High expression of SOX-2 and high expression of podoplanin (Group 2) (n=21)	High expression of SOX-2 and low expression of podoplanin (Group 3) (n=8)	low expression of SOX-2 and high expression of podoplanin (Group 4) (n=11)	X2 (df)	p Value
**Age**							
<40 years	11	2	4	3	2	2.898(3)	0.408
>40 years	49	18	17	5	9		
**Gender**							
Male	47	14	18	7	8	2.092(3)	0.553
Female	13	6	3	1	3		
**Habits**							
Tobacco habits	45	14	18	6	7	5.342 (6)	0.501
No habits	14	6	2	2	4		
**Site**							
Buccal mucosa	20	8	8	3	1	13.388 (15)	0.572
Tongue	22	6	8	3	5		
Other	18	6	5	2	5		
**Stage**							
Early stage	8	0	4	1	3	5.618 (3)	0.132
Advanced stage	51	20	16	7	8		
**Grade**							
Well differentiated	20	3	10	4	3	18.642(6)	.005*
Moderately differentiated	20	4	9	1	6		
Poorly differentiated	20	13	2	3	2		
**Patient outcome**							
Disease free survival	13	2	7	2	2	8.208(6)	0.223
Recurrence	14	4	3	2	5		
Death	3	2	0	1	0		

Follow-up details were not available for 30 cases, The stage of the tumor was unknown for one case and the habit history was unknown for one case

**Table 3:** Association of the expression of SOX2 with the expression of podoplanin

SOX2 expression	Podoplanin expression		X2 (df)	p Value
	Low expression (n=31)	High expression (n=29)		
Low expression (n=29)	21	8	9.675 (1)	0.002
High expression (n=31)	10	21		

**Figure 5:**
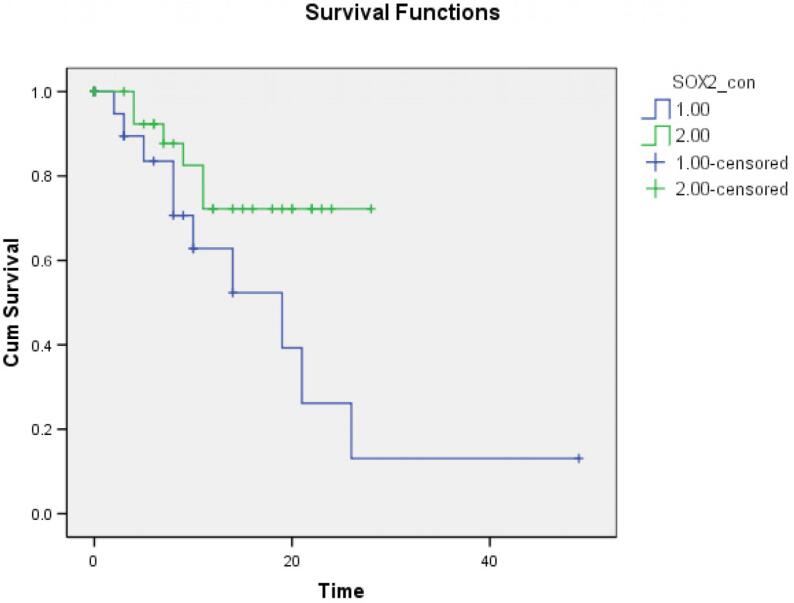
Kaplan–Meier survival plots showing a significant difference in the clinical behavior of the tumors. Tumors with a low expression of SOX2 (blue line) had shorter disease free survival time compared to tumors with a high expression of SOX2 (green line)

Finally, survival analysis was done using the Kaplan-Meier method and log-rank test using the follow-up data of the patients. The patient outcomes are classified as good prognosis (disease free survival) and poor prognosis (cases of recurrence or death). The Kaplan Meier plots showed a significant difference in the clinical behaviour of tumors based on the expression of SOX2 (95% CI – 10.627–27.149, p=0.043, Figure 5). Tumors that showed a high expression of SOX2 had longer disease-free survival periods. However, there was no difference in the clinical behaviour of tumors based on the expression of podoplanin (95% CI 17.525–37.071, p=0.506).

## Discussion

In the recent years, it has been proposed that tumors contain a small population of cells (cancer stem cells) that have gene signatures similar to embryonic stem cells, which drive cancer development, growth and spread.^17^^,^^18^ SOX2, OCT 4, and Nanog are key transcription factors that maintain the self-renewal and pluripotency of embryonic stem cells.^19^ Recently, it has been found that SOX-2 serves as a link between malignancy and “stemness.”^20^

Overexpression of SOX2 in tumor cells is due to amplification of the gene at 3q26.33 region.^21^ The expression of SOX 2 is also regulated by SOX2 core promoter and a number of enhancers located in the upstream and downstream region of SOX2 gene.^22^ In this study a high nuclear expression of SOX2 was evident in 38/60 (63.3%) cases. The overexpression of SOX2 has been reported in 60% to 88% of oral squamous cell carcinoma cases.^5^^,^^12^^,^^23^ The overexpression of SOX2 in tumor cells implies that SOX2 induces stemness in cells that have undergone genotoxic damage.^24^

In this study, a high expression of SOX2 was associated with early stage of the tumor (p=0.029). Likewise, Fu, et al.^19^ (2016) found that a higher SOX2 expression was associated with early stage of the tumor and with smaller tumors, considering that SOX2 played an important role in the early stages of tumorigenesis and that it was an independent prognostic marker.^19^ However, Du, et al.^12^ (2011) found that SOX2 expression was associated with large tumors.

In this study the nuclear expression of SOX2 was associated with higher histopathological grade of the tumor (p=0.002). Most of moderately differentiated tumors 15/20 (75%) showed a positive nuclear expression for SOX-2. He, et al.^25^ (2014) also found a significant association between the tumor grade and the expression of SOX2, and Attarmendal, et al.^5^ (2015) found a higher intensity nuclear expression in tumor cells that were at the invading front. However, Fu, et al.^19^ (2016) did not find any association between the tumor grade and the expression of SOX2. SOX2 plays an essential role in somatic cell reprograming, reversing the epigenetic configuration of differentiated cells back to pluripotent embryonic state.^22^ The properties of cancer stem cells include self-renewal that can result in aberrant differentiation that contributes to cellular heterogeneity.^26^

Yu, et al.^27^ (2016) proposed that there were 3 subpopulations of cancer stem cells in moderately differentiated buccal squamous cell carcinomas that showed varied expression of stem cell markers. The cancer stem cells in the tumor nests both nuclear and cytoplasmic expression of EMA, CD44 Nanog, SOX2, OCT 4 SALL and pSTAT3.^27^ Likewise, Ram, et al.^28^ (2017) also showed that there were 3 subpopulations of cancer stem cells in lip squamous cell carcinomas.

SOX2 can modulate cell aggression and motility by affecting the capacity of migration, invasion and proliferation in tongue squamous cell carcinomas.^23^ Liu, et al.^29^ (2018) reported that SOX2 expression promoted aggressiveness of carcinomas of the tongue through epithelial-mesenchymal transition.^29^ They found that overexpression of SOX2 can also drastically reduce the expression of epithelial markers such as E cadherin, and thereby initiate epithelial-mesenchymal transition. SOX2 promotes tumor metastasis by stimulating epithelial-mesenchymal transition via WNT-β catenin pathway in breast carcinomas.^30^^,^^31^ A recent study by Bayo, et al.^32^ (2015) showed that loss of SOX2 expression induced cell motility via vimentin up regulation in head and neck squamous cell carcinomas.^32^

In this study, Kaplan-Meier analysis showed that tumors with a high expression of SOX2 had better disease-free survival time compared to those with a low-expression of SOX2 (p=0.43). Fu, et al.^19^ (2016) in their study also found that higher levels of SOX2 expression was associated with better disease-free survival even after adjusting the clinicopathological features. Likewise, Attramendal, et al.^5^ (2015) also found that a high nuclear expression of SOX2 at the invasive tumor front was associated with dramatically longer disease-free survival period after post-operative radiotherapy, and they proposed that a high SOX2 expression was indicative of radio-sensitivity of the tumor. Züllig, et al.^13^ (2013) also found that a high expression of SOX2 was associated with an absence of lymph node metastasis and a good prognosis for oral squamous cell carcinomas.

Du, et al.^12^ (2011) and Liu, et al.^28^ (2018), and in their studies found that a high expression of SOX2 was associated with a poor prognosis of tongue squamous cell carcinomas. Du, et al.^12^ (2011) reported that high expression of SOX2 was associated with unfavourable overall, cancer-specific and disease-free survival and was a predictor of recurrence of tongue squamous cell carcinoma.

Podoplanin is a specific marker for lymphatic endothelial cells. The gene for podoplanin is located on chromosome 1p36.21.^33^ The expression of podoplanin in tumor cells is associated with cell migration and invasion.

In this study the expression of podoplanin was evident in 45/60 (75%) cases of oral squamous cell carcinomas. The expression of podoplanin was observed in 82% to 100% of cases of oral squamous cell carcinomas.^14^^,^^34^^,^^35^

Seki, et al.^36^ (2014) found that an overexpression of podoplanin was associate with advanced stage tumors and was also associated with Ki-67 expression, which implies that podoplanin has a role in the growth and progression of oral squamous cell carcinomas^36^. In this study, the association between the stage of the tumor and the expression of podoplanin was not significant (p=0.296). This could be because there were only 8 cases of early stage tumors compared with 51 of advance stage tumors.

In this study, we found a significant association (0.017) between the histological grade of the tumors and the podoplanin expression. Most of the well-differentiated tumors (14/20) showed an overexpression of podoplanin; yet none of the poorly differentiated tumors showed a high podoplanin expression. A similar finding was also seen in Laryngeal squamous cell carcinomas by Rodrigo, et al.^37^ (2010). However, Prasad, et al.^14^ (2015) and Patil, et al.^35^ (2015) found a higher membranous expression of podoplanin in moderately differentiated tumors and Poorly differentiated tumor when compared to well-differentiated tumors. Schacht, et al.^38^(2005) also found a higher expression of podoplanin in moderately differentiated tumors than in well-differentiated tumors.

Podoplanin binds to the ERM (ezrin, radixin and moesin) family of proteins through its cytoplasmic domain, which leads to the activation of small Rho GTPase.^7^^,^^39^ Activation of Rho GTPases mediates alterations in the actin cytoskeleton framework. This induces cell migration and invasion and ectomesenchymal transition.^40^ Additionally, decreased stress fibers and increased filopodia formation in podoplanin positive cells lead to a mesenchymal appearance, which is indicative of epithelialmesenchymal transition.^41^

In addition, podoplanin is also expressed by cancer associated fibroblasts (CAFs). These cells use podoplanin to increase motility and survival of neighbouring tumor cells.^42^

Wicki, et al.^7^ (2007) found that in the absence of E-cadherin switch and EMT, overexpression of podoplanin resulted in increased migration and invasion of cancer cells. Podoplanin is capable of inducing invasion by collective cell and single cell migration. They also found that tumor cells that expressed podoplanin were also positive for the expression of matrix metalloproteinase.^7^

In this study, among the 14 patients with recurrence, 9/14 (64%) cases showed a low expression of podoplanin. However, Kreppel, et al.^43^ (2011) found that a high expression of podoplanin leads to a 3 to 4-fold decrease in the 5-year survival of oral cancer patients.

This study shows a significant association between the expression pattern of SOX2 and Podoplanin (p=.002). Similarly, Saigusa, et al.^44^ (2011), in their investigation on esophageal squamous cell carcinomas, found a significant association between the SOX2 and podoplanin expression. This suggests that cancer cells may acquire stemness properties, which facilitates molecular signaling that leads to invasiveness and metastatic potential of tumor cells. Recently, it was established that SOX2 can also increase the expression of podoplanin in cancer stem cells.^45^

In this study, 7/13 (54%) cases that presented disease-free survival showed a high expression of SOX2 and a low expression of podoplanin. In addition, 5/14 (36%) recurring cases showed a low expression of SOX2 and a high expression of podoplanin. Saigusa, et al.^44^ (2011) evaluated 20 esophageal squamous cell carcinomas cases, following neoadjuvant chemoradiotherapy and found that cases with low expression of both podoplanin and SOX2 had a better prognosis.

Further studies designed to assess the role of stem cell markers in stimulating invasion and metastasis of tumors will help in understanding tumor biology and will be useful in developing effective treatment strategies.

## Conclusion

The study shows that there is an association between SOX2 and podoplanin during the development and progression of oral squamous cell carcinomas. Hence, analysis of the combined expression of SOX-2 and podoplanin can be used to identify the high-risk patients who may benefit from the various adjuvant therapies.
